# Hare Lip and Cleft Palate

**Published:** 1892-06

**Authors:** 


*Hare Lip and Cleft Palate.* By WILLIAM ROSE, F.R.C.S. Pp. xiii., 172. London: H. K. LEWIS. 1891.

The views of Professor Rose on the subject of hare lip and cleft palate ought to be carefully studied by his professional brethren, all classes of whom should find this book interesting and instructive.

Professor Rose deals with the particulars of operative treatment as a surgical artist. Many well-written chapters make up a work which we gladly welcome as a useful and practical addition to our surgical literature.

The volume is well illustrated and carefully indexed.

